# GalNAc in vaginal secretions: a potential non-invasive risk indicator associated with the risk of premature rupture of membranes

**DOI:** 10.1371/journal.pone.0335684

**Published:** 2025-12-02

**Authors:** Lu Wang, Yan Chen, JiaLe Chen, Jie Zhou, Zhen Jia, Yu Chen

**Affiliations:** 1 Wuxi Maternal and Child Health Hospital, Wuxi School of Medicine, Jiangnan University, Jiangsu, China; 2 Hospital Infection Management Section, Wujin Affiliated Hospital of Nanjing University of Traditional Chinese Medicine, Changzhou, Jiangsu, China; 3 Department of Laboratory Haidong Second People’s Hospital, Haidong, Qinghai, China; University of the Punjab Quaid-i-Azam Campus: University of the Punjab, PAKISTAN

## Abstract

Premature rupture of membranes (PROM) is a major contributor to preterm birth and neonatal morbidity. Current diagnostics are predominantly reactive and detect rupture only after it occurs. In a clinical cohort of 60 pregnant women (20 with PROM, 40 gestational controls at 31–36 weeks), we developed and validated a colorimetric assay for N-acetyl-D-galactosamine (GalNAc) in vaginal secretions. GalNAc levels were significantly lower in the PROM group than in controls, and the assay exhibited linearity, precision, stability, and resistance to interference. Receiver-operating characteristic analysis yielded an AUC of 0.821 (95% CI 0.80–0.97). These data support reduced vaginal GalNAc as a potential non-invasive risk indicator of PROM. While longitudinal validation is required, this approach may enable early risk stratification to guide preventive interventions.

## 1. Introduction

Premature rupture of membranes (PROM) is a leading cause of preterm birth and perinatal complications, contributing to 40% of global preterm deliveries and posing significant risks to neonatal health [1]. Current diagnostic methods for PROM, such as amniotic fluid leakage detection and observing fernlike crystal in vaginal fluid, are often invasive and predominantly reactive [2,3].These methods can only confirm membrane rupture after it has occurred and do not provide early warning, limiting the opportunity for timely interventions [4]. The traditional approach to PROM diagnosis relies on detecting amniotic fluid leakage, which is inherently reactive rather than proactive, often resulting in late interventions with limited capacity to alter the course of the condition [5]. There is an urgent need for a predictive, non-invasive biomarker for PROM that can offer early warning and improve clinical outcomes.

Fetal membrane integrity depends critically on the extracellular matrix and epithelial barrier, modulated by structural proteins, glycosaminoglycans, and cell–matrix interactions. Prior studies have documented progressive remodeling of sulfated glycosaminoglycans (GAGs) near term, with more marked changes in PROM pregnancies, suggesting that carbohydrate components may reflect membrane weakening [6]. Separately, glycans at the feto-maternal interface (including O-and N-glycans) influence cell adhesion, immune signaling, and structural stability [7]. Within this milieu, Galectin–glycan circuits (e.g., Galectin-3 binding to GalNAc-containing glycoconjugates) are known to regulate tissue integrity and inflammatory responses in pregnancy biology [8].

Our previous clinical metabolomics profiling of vaginal secretions from a prospective cohort first identified GalNAc depletion as a promising biomarker in women who subsequently developed PROM [9,10]. Mechanistic work in cell and ex vivo systems further showed that GalNAc supplementation modulates inflammation and enhances wound-repair–like responses via the galectin axis [11]. These lines of evidence motivate the present study: we sought to quantitatively validate GalNAc depletion in vaginal secretions, develop a robust colorimetric assay, and assess its performance as a non-invasive risk indicator of PROM.

## 2. Materials and methods

### 2.1. IRB approval

This study was granted ethical approval by the Ethics Committee of Wuxi Maternal and Child Health Hospital (approval number: 2020−01−731−25) and was registered with the China Clinical Trials Registry (registration number: ChiCTR2000034721). All procedures were conducted in accordance with the ethical principles outlined in the Declaration of Helsinki. Written informed consent was obtained from all participants prior to their involvement in the study, ensuring their rights and well-being were protected throughout the research process. The study design and conduct were carefully reviewed and monitored to minimize risks and ensure the ethical integrity of the research.

### 2.2. Prospective cohort study design and sample collection

This validation study was designed to follow up on our initial metabolomics discovery [9]. From a prospective cohort of 300 pregnant women, 60 participants were included in this analysis, comprising 20 PROM cases and 40 matched healthy controls (HC). Sample size was determined using GPower 3.1.9.7 software based on effect sizes observed in our pilot data, which provided >90% power to detect significant differences at α = 0.05.

Vaginal secretion samples were collected from pregnant women at 31–36 weeks of gestation. Sample collection was performed by a trained research midwife using the following standardized procedure: with the pregnant woman in the lithotomy position and after disinfecting the vulva, a disposable sterile speculum was inserted to expose the cervix. A sterile flocked swab was then inserted 3–4 cm into the posterior vaginal fornix, rotated 360° for 10 seconds to ensure adequate sampling of vaginal secretions, and immediately snap-frozen in liquid nitrogen within 5 minutes of collection. All samples were stored at −80°C until batch analysis.

PROM was diagnosed according to the standard guidelines from ACOG Practice Bulletin [12]. Diagnosis required the confirmation of at least two of the following clinical criteria: (1) A clear history of persistent fluid leakage reported by the patient; (2) Sterile speculum examination revealing pooling of amniotic fluid in the posterior vaginal fornix; (3) A positive Nitrazine test (vaginal fluid pH ≥ 7.1); (4) Microscopic observation of ferning. In equivocal cases, the diagnosis was confirmed by detecting insulin-like growth factor-binding protein-1 (IGFBP-1) in vaginal fluid.

### 2.3. Experimental solution preparation method

All chemical reagents were of analytical grade. GalNAc standard (Sigma-Aldrich, USA) and other metabolites were accurately weighed using a microanalytical balance. The GalNAc standard stock solution (4 mg/mL) was prepared by dissolving 8 mg of GalNAc in 2 mL of saturated chloroform aqueous solution. The p-dimethylaminobenzaldehyde reagent was prepared by dissolving 100 mg in 3 mL of acetic acid containing 230 μL of concentrated hydrochloric acid. Sodium carbonate solution (0.3 mol/L) was prepared by dissolving 80 mg in 3 mL of saturated chloroform aqueous solution. For interference studies, mixed solutions containing GalNAc (4 mg/mL) with sucrose, arachidonic acid, or D-alanine (each at 4 mg/mL) were prepared similarly. All solutions were stored at 4°C and protected from light.

### 2.4. Determination of the optimum wavelength and linear standard curve for the full – wavelength ELISA method

We improved the relevant method/process [13].A series of GalNAc standard solutions with different concentrations were prepared. After adding sodium carbonate solution and heating, the solutions were cooled. Then, acetic acid, p-dimethylamino benzaldehyde reagent, and acetic acid were added again. A full-wavelength ELISA scan was performed in the wavelength range of 400–700 nm to determine the optimal wavelength. The linear standard curve was plotted using the Optical Density(OD) value as the vertical coordinate and the GalNAc content as the horizontal coordinate.

### 2.5. Accuracy, repeatability, and stability tests

To evaluate the reliability of the measurement, precision, repeatability, and stability tests were conducted. In the precision test, the same concentration of heated GalNAc solution was measured six times. In the repeatability test, six portions of sample solution and 1 mg/mL standard solution were measured. In the stability test, the mixed solution was observed, and the OD value was measured every 20 minutes within 0–120 minutes.

### 2.6. Vaginal swab elution test

Vaginal swabs were collected and preserved in GalNAc standard solution and stored in a liquid nitrogen tank and a −80°C refrigerator. The swabs were cut and eluted with different volumes of saturated chloroform aqueous solution and pure water. After resting for different periods, the swabs were centrifuged at different speeds and times, and the filtrate was collected for color development and OD value determination.

### 2.7. Interference of other metabolites with color intensity

Considering the potential influence of vaginal metabolites on color intensity, representative substances of vaginal metabolites (sucrose), lipids (arachidonic acid), and amino acids (D–alanine) were added to a known concentration of GalNAc to determine their interference with color intensity.

### 2.8. Analysis of galectin-3 expression in amniotic tissues

To investigate the potential mechanism linking GalNAc depletion to PROM pathogenesis, we analyzed the expression of galectin-3, a key GalNAc-binding protein. Amniotic membrane tissues were collected immediately after delivery under sterile conditions. Tissues were washed with phosphate-buffered saline (PBS, pH 7.4) and homogenized in RIPA lysis buffer containing protease inhibitors. The total protein concentration was determined using the bicinchoninic acid (BCA) assay. Galectin-3 levels were quantified using a commercial human Galectin-3 ELISA kit (R&D Systems, USA, catalog# DGAL30) according to the manufacturer’s protocol. All samples were analyzed in duplicate, and results were normalized to total protein content.

### 2.9. Data processing and statistical analysis

Data analysis was conducted using SPSS 22.0 (IBM Corp., Armonk, NY) and R 4.2.2 (R Foundation for Statistical Computing, Vienna, Austria). Continuous variables were tested for normality using the Shapiro-Wilk test. Normally distributed data were expressed as mean ± standard deviation and compared using independent samples t-test, while non-normally distributed data (including GalNAc levels) were expressed as median (interquartile range) and compared using Mann-Whitney U test. Categorical variables were compared using Chi-square test or Fisher’s exact test as appropriate.

The diagnostic performance of GalNAc was evaluated by Receiver Operating Characteristic (ROC) curve analysis, with the area under the curve (AUC) and its 95% confidence interval (CI) calculated using the DeLong method. The optimal cut-off value was determined by maximizing the Youden index. Statistical significance was set at two-sided *p* < 0.05. Power analysis was performed using GPower 3.1.9.7, confirming adequate statistical power (>90%) for the observed effect sizes.

## 3. Results

### 3.1. Development and optimization of a full-wavelength colorimetric assay for GalNAc quantification

**Fig 1:**
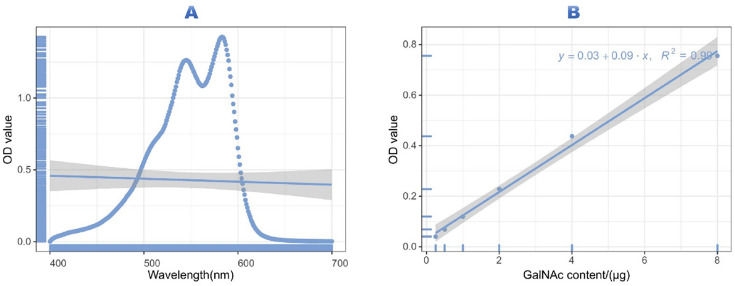
Determination of optimum wavelength and linear equation by colorimetric method. (A) presents the wavelength-OD graph generated from the full-wavelength ELISA scan between 400–700 nm. (B) shows the linear equation derived as y = 0.09x+ 0.03 (R² = 0.99), indicating a strong linear relationship between the measured OD values and GalNAc concentrations. These results confirm the reliability of the assay for quantifying GalNAc levels in clinical samples.

We developed a quantitative colorimetric assay based on the specific reaction between GalNAc and p-dimethylaminobenzaldehyde. Through systematic optimization, we identified the maximum absorbance at 583 nm (Fig 1A). The assay demonstrated excellent linearity across the concentration range of 0.25–4.0 mg/mL with the regression equation y = 0.09x + 0.03 (R² = 0.99, *p* < 0.001) (Fig 1B).

### 3.2. Analytical validation of the GalNAc assay

**Fig 2:**
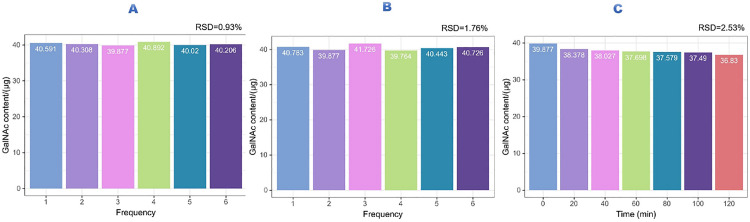
Assay performance evaluation. (A) Illustrates the precision test results of the GalNAc assay, with a relative standard deviation (RSD) of 0.93%. (B) displays the repeatability test outcomes, showing an RSD of 1.76%. (C) presents the stability evaluation, with an RSD of 2.53% over 120 minutes. These findings demonstrate the robustness and reliability of the assay in measuring GalNAc levels over time.

The colorimetric assay exhibited outstanding analytical performance. Precision testing showed low variability with intra-assay and inter-assay relative standard deviations (RSD) of 0.93% and 1.76%, respectively (Fig 2A, B). Stability assessment confirmed that the reaction mixture remained stable for at least 120 minutes with RSD < 3% (Fig 2C).

### 3.3. Optimization of vaginal swab processing and interference assessment

**Fig 3:**
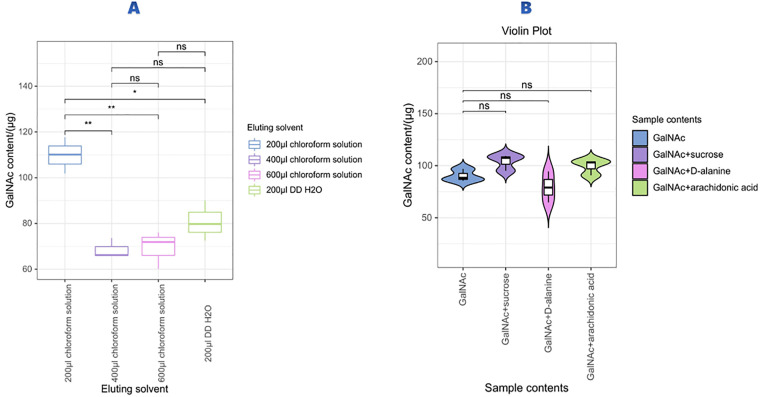
Vaginal swab elution optimization and interference analysis. (A)shows the elution efficiency of different volumes of saturated chloroform aqueous solution used for vaginal swab elution. (B) shows the colorimetric assay results of GalNAc in the presence of sucrose, with a P value of 0.0899. These results indicate that these metabolites do not significantly interfere with the GalNAc assay, ensuring accurate measurement of GalNAc levels.

We established an optimized protocol for processing vaginal swabs to maximize GalNAc recovery. The optimal conditions were determined as: one elution using 200 μL saturated chloroform aqueous solution, 1 hour shaking at 4°C, and centrifugation at 15,000 rcf for 5 minutes, achieving recovery rates exceeding 90% (Fig 3A).

Interference testing revealed that common vaginal metabolites including sucrose (*p =* 0.0899), arachidonic acid (*p =* 0.1820), and D-alanine (*p =* 0.2012) did not significantly affect GalNAc detection (Fig 3B), demonstrating the assay’s specificity in complex biological matrices.

### 3.4. GalNAc levels in vaginal secretions and diagnostic performance for PROM

The baseline characteristics of the study participants are summarized in Table 1. No significant differences were observed in maternal age (30.0± 1.9 *vs*. 31.1 ± 3.2 years, *p* =  0.13) or white blood cell count (9.5 ± 2.9 vs. 10.5 ± 1.3 × 10⁹/L, *p* = 0.52) between the control and PROM groups, indicating well-matched cohorts for these potential confounders.

**Table 1:** Baseline characteristics of study participants.

Variable	HC_Group	PROM_Group	*p_*value
**Maternal Age (years)**	30.0 ± 1.9	31.1 ± 3.2	0.13
**White Blood Cell Count (×10**⁹**/L)**	9.5 ± 2.9	10.5 ± 1.3	0.52

**Fig 4:**
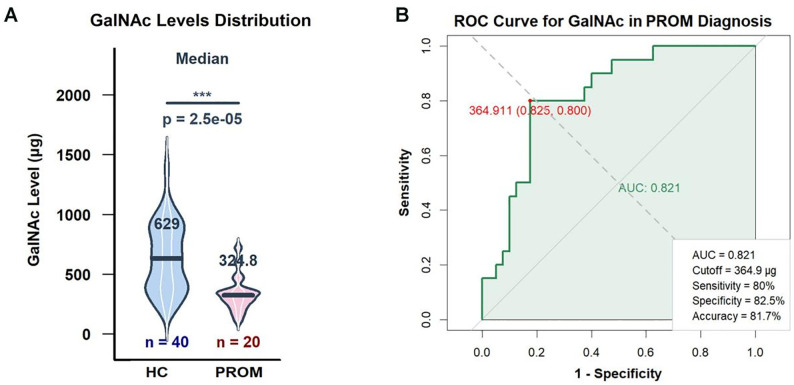
Vaginal swab elution optimization and interference analysis. GalNAc quantification in vaginal secretions and its association with PROM risk. (A) GalNAc levels measured by colorimetric assay in vaginal swabs from healthy controls (n = 40) and PROM cases (n = 20). Data are presented as median with interquartile range. *p < 0.001, Mann-Whitney U test.(B) ROC curve analysis of GalNAc levels for discriminating PROM cases from healthy controls. The area under the curve (AUC) was 0.821 (95% CI: 0.80–0.97). The optimal cut-off value of 364.911μg provided 80.0% sensitivity and 82.5% specificity.

Analysis of 60 clinical samples (20 PROM, 40 controls) revealed significantly reduced GalNAc levels in the PROM group (median 324.8 μg, IQR: 245.6–418.9) compared to controls (median 629.0 μg, IQR: 558.3–745.2; *p *= 2.5 × 10 ⁻ ⁵) (Fig 4A).

ROC analysis demonstrated excellent diagnostic performance with an AUC of 0.821 (95% CI: 0.80–0.97; *p *< 0.001) (Fig 4B). The optimal cut-off value of 364.911 μg provided 80.0% sensitivity and 82.5% specificity for PROM identification.

### 3.5. Detection of GalNAc receptors in amniotic tissue—Galectin-3

**Fig 5:**
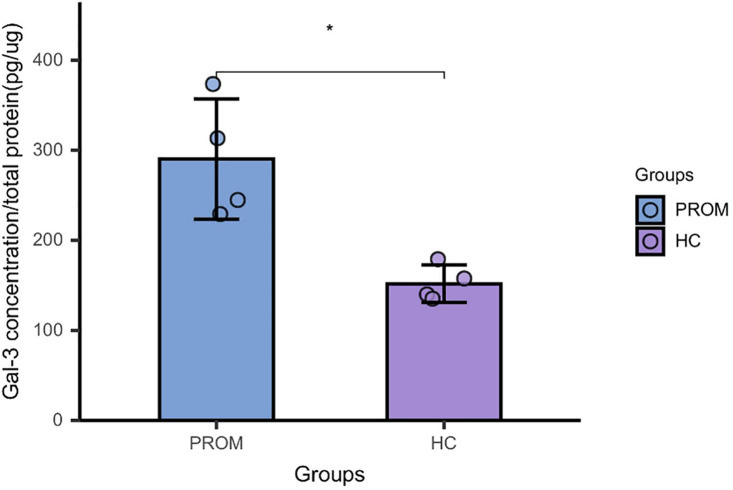
Detection of GalNAc receptors in amniotic tissue-Galectin-3. Galectin-3, a GalNAc receptor, exhibited lower expression in amniotic tissue from the PROM group (*p* = 0.0277), suggesting a potential link between GalNAc levels and membrane integrity. The downregulation of galectin-3 in PROM cases aligns with the hypothesis that GalNAc depletion may lead to a weakened glycocalyx layer, thereby increasing the susceptibility of fetal membranes to rupture.

Consistent with the observed GalNAc depletion in vaginal secretions, galectin-3 protein expression was significantly downregulated in amniotic tissues from the PROM group compared to controls (*p* = 0.0277) (Fig 5), suggesting a potential mechanistic link between GalNAc availability and membrane integrity through the galectin-3 pathway.

## 4. Discussion

This study provides quantitative evidence that reduced vaginal GalNAc concentrations are significantly associated with premature rupture of membranes (PROM), supporting a biochemical mechanism in which glycocalyx degradation contributes to membrane fragility. The validated colorimetric assay demonstrated strong analytical performance, high reproducibility, and clinical discriminative power, suggesting that GalNAc detection in vaginal secretions could serve as a non-invasive and clinically deployable early biomarker for PROM risk assessment.

Mechanistically, GalNAc is a critical monosaccharide component in O-linked glycosylation, serving as the structural basis for mucin-type glycans that maintain epithelial integrity and hydration. Reduced GalNAc availability likely disrupts the glycocalyx architecture, impairing mucosal barrier properties and promoting premature weakening of fetal membranes. Recent molecular studies have revealed that the GalNAc-galectin axis plays a pivotal role in maintaining epithelial–mesenchymal homeostasis and regulating local immune tolerance [14,15]. Specifically, galectins-particularly galectin-3-recognize GalNAc-containing glycoconjugates, mediating cell-matrix adhesion and anti-inflammatory signaling. Depletion of these glycans reduces galectin-3 binding capacity and promotes extracellular matrix disorganization, rendering the amniochorionic membranes more susceptible to rupture.

Our observation of decreased galectin-3 expression in amniotic tissues from PROM cases aligns with this mechanistic framework. GalNAc enhanced epithelial repair and upregulated galectin-3 and galectin-1 expression in trophoblast cells, promoting tissue healing and barrier restoration [14]. Likewise, Boron et al. found differential expression of galectin-1 and galectin-9 in placentas from PPROM pregnancies, further underscoring the broad relevance of galectin family members in maintaining membrane integrity [15].

Beyond PROM, dysregulation of galectins has been implicated in other pregnancy disorders, such as preeclampsia, where placental galectin-3 levels are markedly reduced and correlate with impaired trophoblast invasion [16]. These convergent findings suggest that galectin deficiency-whether secondary to altered glycosylation or local inflammation-may represent a common pathological denominator across gestational complications involving barrier dysfunction.

At the cellular level, galectins orchestrate epithelial–mesenchymal transition (EMT) processes during tissue remodeling and wound healing. Recent work by Perez-Moreno E et al. demonstrated that galectin-mediated glycan recognition modulates EMT transcription factors and extracellular matrix turnover, supporting tissue repair but also influencing fibrosis and mechanical resilience [17]. This aligns with our hypothesis that GalNAc depletion and galectin-3 loss collectively weaken the membrane’s structural framework and reparative potential.

From a clinical perspective, current PROM diagnostics rely on detection of amniotic fluid proteins (e.g., IGFBP-1, PAMG-1), which identify rupture only after it has occurred. In contrast, GalNAc quantification offers a predictive biochemical signature that could detect subclinical membrane vulnerability before rupture, allowing timely intervention. Integrating such assays with existing prenatal screening workflows could substantially improve early-risk stratification.

## 5. Conclusions

In conclusion, our findings propose a novel perspective in which GalNAc-related glycocalyx remodeling serves as a biochemical precursor and predictive marker for PROM, acting upstream of known inflammatory and proteolytic cascades. Although the exact molecular mechanisms remain to be elucidated, the convergence of glycosylation biology and reproductive immunology offers a promising avenue for early, non-invasive risk prediction and preventive intervention in PROM.

## Supporting information

S1 Data Dataset. (XLSX)

## Abbreviations

PROM: Premature rupture of membrane
GalNAc: N-acetyl-D-galactosamine
HC: Healthy control
PBS: P-dimethylamino benzaldehyde solution
OD: Optical Density
BCA: Bicinchoninic Acid Assay
ELISA: Enzyme-linked immunosorbent assay
SPSS: Statistical Package for the Social Sciences
RSD: relative standard deviation
WBC: white blood cell
HR: hazard ratio
CI: confidence interval
